# Supplementary material for: mHealth intervention (mTB-Tobacco) for smoking cessation in people with drug-sensitive pulmonary tuberculosis in Bangladesh and Pakistan: protocol for an adaptive design, cluster randomised controlled trial (Quit4TB)
Source: BMJ Open. 2025 Feb 25;15(2):e089007. doi: 10.1136/bmjopen-2024-089007 (PMC12083406; doi:10.1136/bmjopen-2024-089007)
Supplement: online supplemental figure 1 [file bmjopen-15-2-s001.pdf]

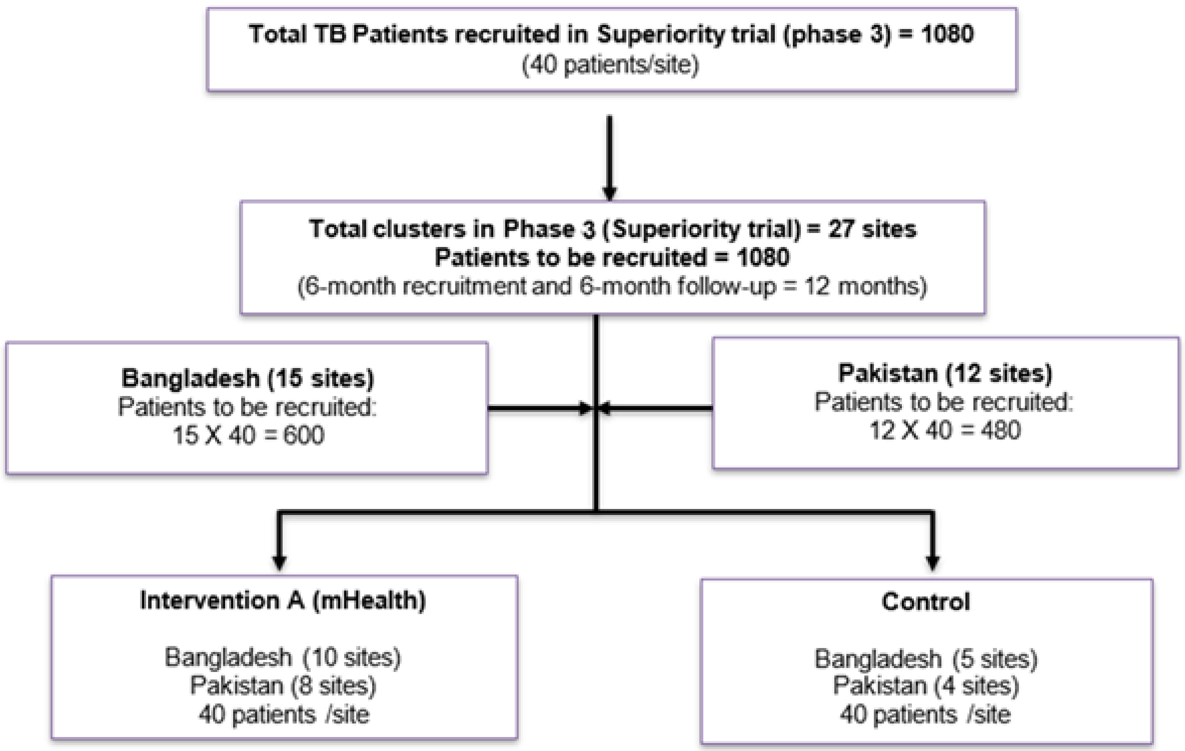

Supplementary Figure 1a: Cluster numbers and cluster sizes for the superiority trial in Pakistan and Bangladesh

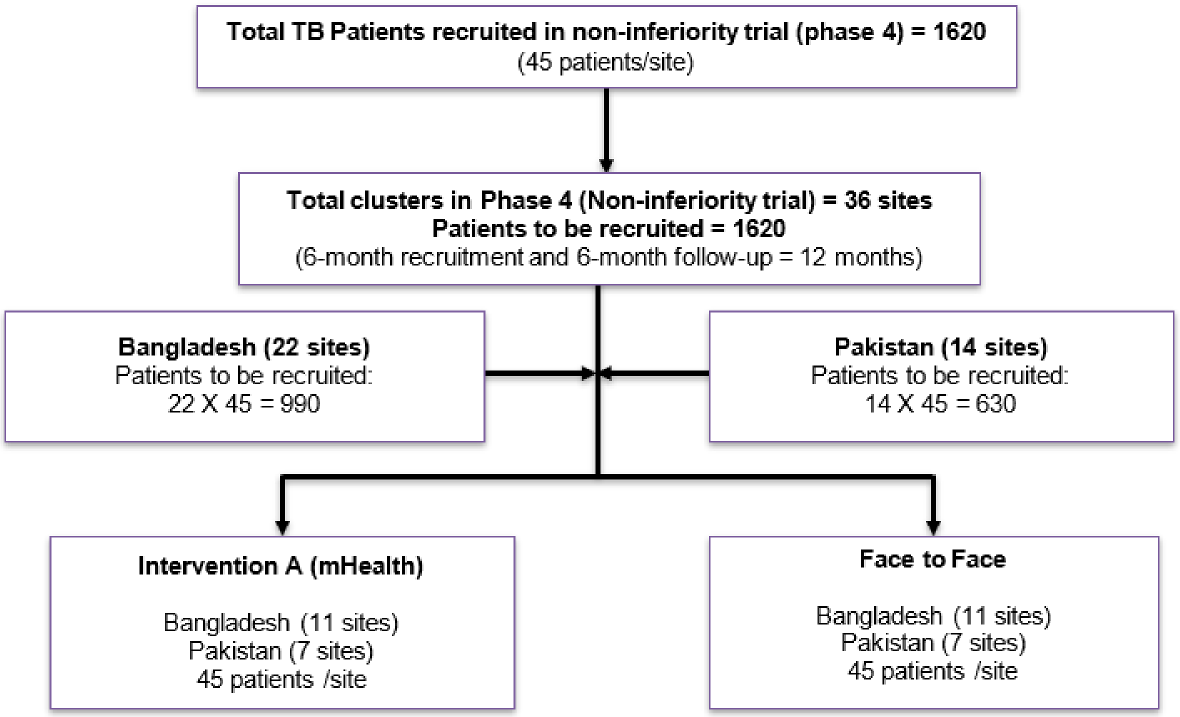

Supplementary Figure 1b: Cluster numbers and cluster sizes for the non-inferiority trial in Pakistan and Bangladesh
