# Supplementary material for: mHealth intervention (mTB-Tobacco) for smoking cessation in people with drug-sensitive pulmonary tuberculosis in Bangladesh and Pakistan: protocol for an adaptive design, cluster randomised controlled trial (Quit4TB)
Source: BMJ Open. 2025 Feb 25;15(2):e089007. doi: 10.1136/bmjopen-2024-089007 (PMC12083406; doi:10.1136/bmjopen-2024-089007)
Supplement: online supplemental file 1 [file bmjopen-15-2-s003.pdf]

**Participant Information Sheet (Adults)**
**(Phase 3 mTB-Tobacco with Usual Care)**

# A mHealth intervention (mTB-Tobacco) for smoking cessation in people with tuberculosis: a two-stage adaptive design, randomised trial
# Quit 4 TB Trial

You are invited to take part in a research study. To help you decide whether or not to take part, it is important for you to understand why the research is being done and what it will involve. Please take time to read the following information carefully. Talk to others about the study if you wish. Contact us if there is anything that is not clear, or if you would like more information. Take time to decide whether or not you wish to take part.

## 1. What is Quit 4 TB Trial?

Quit 4 TB Trial is a research study on tobacco smoking among tuberculosis (TB) patients and we are inviting you to take part in this study. TB is an infectious disease caused by bacteria that usually attacks your lungs but can also affect any part of the body, including bone, brain and nervous system. It is spread when a person inhales tiny droplets from the coughs or sneezes of an infected person. More than two thousand TB patients in Bangladesh and Pakistan are invited to participate in the Quit 4 TB trial. Here, we explain why we are doing Quit 4 TB trial and what it will involve. This will help you to decide whether to participate in this study. You are free to choose whether or not to participate in this study.

## 2. Why are we doing a Quit 4 TB Trial?

Tobacco smoking is bad for health, particularly of those with TB. If TB patients continue to smoke during their treatment they may not recover from it. Quit 4 TB trial is aimed to test how best to get TB patients to stop smoking and to successfully complete TB treatment. However, tobacco smoking is highly addictive and smokers find it difficult to give up. We have designed a mTB-Tobacco programme in which we will send motivational and informative short message service (SMS) messages throughout a patient's TB treatment.

## 3. Who is doing this study?

The study is being done by Professor Rumana Huque, ARK Foundation in Bangladesh and Dr Amina Khan, The Initiative in Pakistan, in collaboration with the University of Edinburgh. The study has been funded by The NIHR Global Health Research Unit on Respiratory Health (RESPIRE) and is sponsored by University of Edinburgh in the United Kingdom. The study has been approved by the ethics committees of the University of Edinburgh Medical School Research Ethics Committee (EMREC), in addition to the Bangladesh Medical Research Council and the National Bioethics Committee PMRC in Pakistan.

## 4. Do I have to take part?

No, it is up to you to decide whether or not to take part. If you do decide to take part you will be given this information sheet to keep and be asked to sign a consent form, which will be counter-signed by the researcher conducting this study. If you decide to take part you are still free to withdraw at any time and without giving a reason. Deciding not to take part or withdrawing from the study will not affect the healthcare that you receive, or your legal rights.

## 5. What will I have to do?

This study is a randomised control trial (RCT). This is a method where participants are put into groups and each group is given a treatment and the results are compared to see if one is better. Each participating clinic will be randomly allocated into a group by chance. If you agree to participate in the Quit 4 TB trial, the treatment that you receive will be the one in which your health centre is allocated to. Patients who attend the health centre that is allocated to mTB-Tobacco group will be given access to the mHealth smoking cessation package. mHealth is a general term for the use of mobile devices (e.g. mobile phones and other wireless technology) in medical care and public health services. mTB- Tobacco programme delivers SMS messages via mobile phones to TB patients. Participants in this group must have the access to a personal mobile phone to receive SMS content.

> We will send you a total of 178 SMS messages over a period of 6 months to help you quit smoking and complete your TB treatment effectively. In the first two months, the frequency of messages would be 4 to 5 messages per day, in next two month the frequency would reduce to 1 to 2 messages per day and in the last two months there will be 1 message sent per week.

In addition to mHealth smoking cessation package, this group will also receive education leaflets from TB health professionals. Patients who attend the health centre that is allocated to standard usual care will receive only education leaflets.

- **<u>Urine sample</u>**

For participants who use smokeless tobacco, we would like you to provide urine sample at month 6 in order to check for nicotine in your urine. Urine sample will be collected from these participants on their follow up visit. The participants will be provided with containers and private premises to provide the sample. Nicotine dipstick test will be carried out by the research assistants on the sample and result recorded. The sample will then be discarded.

- **Assessments**

Taking part in the study will involve attending your usual visits to the clinic. You will meet the research team on three separate occasions during your routine planned clinic appointments to complete some assessments at the start, 9 weeks later and 6 months later. At each occasion, we would like to complete a questionnaire about you, your health, well-being, smoking habits, and healthcare expenses. This takes around 60 minutes of your time on the first visit and about 20 minutes on subsequent visits. A researcher assistant will complete the questionnaires together with you. At month 6 we would also like you to breathe into an instrument, which will assess your smoking. It is important for the study that you try to complete all the follow-up visits. We would also need to obtain information from your health records, about the medicines you have been taking and your recovery from TB. We need to collect information about your other medical conditions and healthcare costs so that we can compare the costs and outcomes of healthcare treatments. You will also receive a weekly call by the research assistant (on provided number) to ask about SMS messages being received, and to remind about follow up visits. You will receive travel reimbursement for two follow up visits including week-9 and 6-month follow up. If we need to arrange extra follow-up visits to review your progress, after week 9 and after 6-month, we will let you know and reimburse the travel expenses to extra visits as well. You will be reimbursed 400PKR/visit.

**6. Are there any risks or disadvantages?**

There are no potential risks/harms associated in taking part in this study. You will have to make time to answer the questions on your regular visits to the TB centre.

**7. What are the benefits of taking part?**

We will provide you some advice on how to quit smoking and successfully complete TB treatment. You will find the advice helpful in stopping smoking and will be beneficial for the whole society.

**If I take part, will my information be kept confidential?**

All the information we collect from you will be kept confidential on secure computers. Your identity and location in this study will remain strictly confidential. The results of the study, including data, may be published for scientific purposes but will not give your name or include any identifiable references to you.

All the information we collect during the course of the research will be kept confidential and there are strict laws which safeguard your privacy at every stage.

**How will we use information about you?**

We will need to collect the following personal identifiable information from you for this research study:

- Name (e.g. for Consent Purposes)
- Contact details (mobile number, address and CNIC number)
- Socio-demographic data
- Self-reported data on tobacco use behavior (past and present)

Any information that contains your name and personal identifiers will be removed so that you cannot be identified. All information will be coded and de-identified. The information we have collected in paper copies will be stored under lock and key cabinet at

The Initiative,
Main Office
Orange Grove Farm, Main Korung Rd
Banigala, Islamabad
Pakistan

AKR Foundation
Suite C-3 & C-4,
House #06,
Road # 109,
Gulshan-2, Dhaka-1212,
Bangladesh

The electronic data will be stored in a secured computer which can only be accessed with a secure password. Only the researchers will have access to the data.

All of your personal data including identifying information will be stored in a secure data-storing facility and secure servers for a minimum of 10 years following the end of the study at

The Initiative,
Main Office
Orange Grove Farm, Main Korung Rd
Banigala, Islamabad
Pakistan

AKR Foundation
Suite C-3 & C-4,
House #06,
Road # 109,
Gulshan-2, Dhaka-1212,
Bangladesh

Data will be transferred from Bangladesh and Pakistan to University of Edinburgh in the UK via a secure, password-protected online platform (DataSync) and then stored on a secure, password-protected DataVault / DataShare at University of Edinburgh for a minimum of 10 years following the end of the study. Your de-identified data will be shared with other researchers for future use. DataShare is an open access repository for anonymised data, which means that all non-identifiable data is freely available. DataVault is a secure repository for sensitive information which can only be accessed by approved researchers who have undergone a rigorous application and review process. After 10 years, all paper records will be shredded and disposed of securely and electronic records will be permanently erased. The data will be archived for up to 10 years on the University of Edinburgh server.
If you have any concerns about how we will use your information, please contact:

- The University of Edinburgh Data Protection Officer: dpo@ed.ac.uk
- Contact the Principal Investigator
    - Amina Khan, The Inititative, Main Office, Orange Grove Farm, Main Korung Road, Banigala, Islambad, Pakistan
    - Rumana Huque, ARK Foundation, Suite C-3 & C-4, House # 06, Road # 109, Gulshan-2, Dhaka-1212, Bangladesh

**8. How will the results be reported?**

The results of the trial will be published in international journals and presented at national and international meetings in both TB care and Tobacco Control. A lay summary of results will be distributed to local TB care networks. You will not be identifiable from any published results.

**9. What will happen if I want to stop?**

The project is voluntary so if you decide that you no longer want to carry on, you can stop at any time. You don't have to give a reason if you do this and this will not affect your treatment at the hospital in any way or your legal rights. We would not ask for any further information but still like to use the information you have already provided in our research. However, if you specifically ask us not to use this information, we will delete this from our records. This will not affect your TB treatment. You will not be able to withdraw use of data from results that have already been published.

**10. What happens next?**

Any further questions you have about this study will be answered by the Principal Investigators:

| | |
|---|---|
| Dr Amina Khan<br>The Initiative Islamabad Pakistan<br>Phone: 051-8732651<br>Email: aminakhan67@gmail.com. | Professor Rumana Huque<br>ARK Foundation, Bangladesh<br>Phone: +88 02 55069866<br>Email: rumanah14@yahoo.com |

If you are ready to participate, we can continue now or if you wish to take 24 hours to consider participating in the study, we can schedule another appointment.

**11. Who do I contact if I have a problem or I have questions about the study?**

If you wish to discuss any aspect of the trial or have any concerns, the Principal Investigators are available to provide a complete explanation:

| | |
|---|---|
| Dr Amina Khan<br>The Initiative Islamabad Pakistan<br>Phone: 051-8732651<br>Email: aminakhan67@gmail.com. | Professor Rumana Huque<br>ARK Foundation, Bangladesh<br>Phone: +88 02 55069866<br>Email: rumanah14@yahoo.com |

**12. Independent Contact Details**

If you would like to discuss this study with someone independent of the study please contact

| | |
|---|---|
| Dr Faiza Aslam<br>Director RGMO<br>Rawalpindi Medical University, Pakistan<br>Email: drfaizaaslam@gmail.com | Dr. Khaleda Islam<br>Technical Adviser<br>The Flemming Fund Bangladesh<br>Email: dr.khaleda.islam@gmail.com |

**Participant Consent Form (Adult)**
**(Phase 3 mTB-Tobacco with Usual Care)**
**A mHealth intervention (mTB-Tobacco) for smoking cessation in people with tuberculosis: a two-stage adaptive design, randomised trial**
**Quit 4 TB Trial**

To be completed by the patient

If you agree with the statements, please put an initial in the boxes below.

| | |
|---|---|
| 1. I confirm that I have read and understood the Participant Information Sheet for version v3.0 dated 28th March 2023 and have had the opportunity to consider the information, ask questions and have had these questions answered satisfactorily. | |
| 2. I understand that my participation is voluntary and that I am free to withdraw at any time, without giving any reason and without my medical care and/or legal rights being affected. | |
| 3. I understand that by taking part in this study my identity and location will be kept confidential. | |
| 4. I understand that the information I share and the data from my routine TB care will be accessed by the researchers. I give permission for these individuals to have access to my records. | |
| 5. I understand that data generated during the study will be sent outside of my home country to the United Kingdom, where laws protecting my personal information may be different to my own country. | |
| 6. I understand that de-identified data from this study may be shared with other researchers for future use. | |
| 7. I understand that my name will not be linked with the research materials, and I will not be identified or identifiable in any reports that result from the research. | |
| 8. I understand that if I withdraw from the study, the information I have already provided will still be used for the research. However, if I specifically ask you not to use this information, you will delete this from your records. | |
| 9. I agree to provide my complete contact details including CNIC number. | |
| 10. I understand that if I have concerns about this research, I can contact the Principal Investigator. | |
| 11. I agree to take part in the above titled study. | |

Participant's Full Name ____________________________________________

Date ______________________________________

| |
|---|
| Participant 's signature/thumb impression to be used if participant is illiterate: |
| Participant's CNIC Number: ___________________________________________ |
| Participant's Address: _________________________________________________________________________<br>Telephone #: ____________________ |
| **Name of Person Obtaining Consent:**<br>Researcher Signature ___________________ Name of the Researcher_______________________<br>Date ___________________ |

**Name of Witness:**
Signed by impartial literate third party witness (*In case of illiterate Participant/ Legally Authorized Representative is illiterate*)

Signature:

Date: Place:

An NIHR Global Health Research Unit on Respiratory Health (RESPIRE) at the University of Edinburgh project.
***www.ed.ac.uk/usher/respire***
*This research was funded by the UK National Institute for Health and Care Research (NIHR) (Global Health Research Unit on Respiratory Health (RESPIRE); NIHR132826) using UK aid from the UK Government to support global health research.*
*The views expressed in this publication are those of the author(s) and not necessarily those of the NIHR or the UK Government*

**Participant Information Sheet (Adult)**
**(Phase 4: mTB-Tobacco with Behavioral support)**
**A mHealth intervention (mTB-Tobacco) for smoking cessation in people with tuberculosis: a two-stage adaptive design, randomised trial**
**Quit 4 TB Trial**

**You are invited to take part in a research study. To help you decide whether or not to take part, it is important for you to understand why the research is being done and what it will involve. Please take time to read the following information carefully. Talk to others about the study if you wish. Contact us if there is anything that is not clear, or if you would like more information. Take time to decide whether or not you wish to take part.**

**1. What is Quit 4 TB Trial?**

Quit 4 TB Trial is a research study on tobacco smoking among tuberculosis (TB) patients and we are inviting you to take part in this study. TB is an infectious disease caused by bacteria that usually attacks your lungs but can also affect any part of the body, including bone, brain and nervous system. It is spread when a person inhales tiny droplets from the coughs or sneezes of an infected person. More than two thousand TB patients in Bangladesh and Pakistan are invited to participate in the Quit 4 TB trial. Here, we explain why we are doing Quit 4 TB trial and what it will involve. This will help you to decide whether to participate in this study. You are free to choose whether or not to participate in this study.

**2. Why are we doing a Quit 4 TB Trial?**

Tobacco smoking is bad for health, particularly of those with TB. If TB patients continue to smoke during their treatment they may not recover from it. Quit 4 TB trial is aimed to test how best to get TB patients to stop smoking and to successfully complete TB treatment. However, tobacco smoking is highly addictive and smokers find it difficult to give up. We have designed a mTB-Tobacco programme in which we will send motivational and informative short message service (SMS) messages throughout a patient's TB treatment.

**3. Who is doing this study?**

The study is being done by Professor Rumana Huque, ARK Foundation in Bangladesh and Dr Amina Khan, The Initiative in Pakistan, in collaboration with the University of Edinburgh. The study has been funded by The NIHR Global Health Research Unit on Respiratory Health (RESPIRE) and is sponsored by University of Edinburgh in the United Kingdom. The study has been approved by the ethics committees of the University of Edinburgh Medical School Research Ethics Committee (EMREC), in addition to the Bangladesh Medical Research Council and the National Bioethics Committee PMRC in Pakistan.

**4. Do I have to take part?**

No, it is up to you to decide whether or not to take part. If you do decide to take part you will be given this information sheet to keep and be asked to sign a consent form, which will be counter-signed by the researcher conducting this study. If you decide to take part you are still free to withdraw at any time and without giving a reason. Deciding not to take part or withdrawing from the study will not affect the healthcare that you receive, or your legal rights.

**5. What will I have to do?**

This study is a randomised control trial (RCT). This is a method where participants are put into groups and each group is given a treatment and the results are compared to see if one is better. Each participating clinic will be randomly allocated into a group by chance. If you agree to participate in the Quit 4 TB trial, the treatment that you receive will be the one in which your health centre is allocated to. Patients who attend the health centre that is allocated to mTB-Tobacco group will be given access to the mHealth smoking cessation package. mHealth is a general term for the use of mobile devices (e.g. mobile phones and other wireless technology) in medical care and public health services. mTB- Tobacco programme delivers SMS messages via mobile phones to TB patients. Participants in this group must have the access to a personal mobile phone to receive SMS content. We will send you a total of 178 SMS messages over a period of 6 months to help you quit smoking and complete your TB treatment effectively. In the first two months, the frequency of messages would be 4 to 5 messages per day, in next two month the frequency would reduce to 2 to 3 messages per day and in the last two month there will be 1 to 2 messages sent per week. Patients who attend the health centre that is allocated to face-to-face behavioural support will receive two face-to-face sessions delivered at day 0 and day 5 (+2) and last 10 and 5 minutes respectively.

Urine sample

For participants who use smokeless tobacco, we would like you to provide urine sample at month 6 in order to validate check for nicotine in your urine. Urine sample will be collected from these participants on their follow up visit. The participants will be provided with containers and private premises to provide the sample. Nicotine dipstick test will be carried out by the research assistants on the sample and result recorded. The sample will then be discarded.

Assessments

Taking part in the study will involve attending your usual visits to the clinic. You will meet the research team on three separate occasions during your routine planned clinic appointments to complete some assessments at the start, 9 weeks later and 6 months later. At each occasion, we would like to complete a questionnaire about you, your health, well-being, smoking habits, and healthcare expenses. This takes around 60 minutes of your time on the first visit and about 20 minutes on subsequent visits. A researcher assistant will complete the questionnaires together with you. At month 6 we would also like you to breathe into an instrument, which will assess your smoking. It is important for the study that you try to complete all the follow-up visits. We would also need to obtain information from your health records, about the medicines you have been taking and your recovery from TB. We need to collect information about your other medical conditions and healthcare costs so that we can compare the costs and outcomes of healthcare treatments

You will not receive travel reimbursement because the assessments will be carried out during your visits to the clinic for your usual care review with the doctor. If we need to arrange follow-up visits to review your progress, we will arrange for you to come and see us at the hospital. These extra visits will happen after week 9 and after 6 month. These extra visits will happen after week 9 and six month. To cover your travel expenses for these extra visits, you will be reimbursed 200PKR/visit or 113BDT/visit (about £0.97/visit).

**6. Are there any risks or disadvantages?**

There are no potential risks/harms associated in taking part in this study. You will have to make time to answer the questions on your regular visits to the TB centre.

**7. What are the benefits of taking part?**

We will provide you some advice on how to quit smoking and successfully complete TB treatment. You will find the advice helpful in stopping smoking and will be beneficial for the whole society.

If I take part, will my information be kept confidential?

All the information we collect from you will be kept confidential on secure computers. Your identity and location in this study will remain strictly confidential. The results of the study, including data, may be published for scientific purposes but will not give your name or include any identifiable references to you.

All the information we collect during the course of the research will be kept confidential and there are strict laws which safeguard your privacy at every stage.

**How will we use information about you?**

We will need to collect the following personal identifiable information from you for this research study:

- Name (e.g. for Consent Purposes)
- Contact details (mobile number, address and CNIC number)
- Socio-demographic data
- Self-reported data on tobacco use behavior (past and present)

Any information that contains your name and personal identifiers will be removed so that you cannot be identified. All information will be coded and de-identified. The information we have collected in paper copies will be stored under lock and key cabinet at

The Initiative,
Main Office
Orange Grove Farm, Main Korung Rd
Banigala, Islamabad
Pakistan
AKR Foundation
Suite C-3 & C-4,
House #06,

Road # 109,
Gulshan-2, Dhaka-1212,
Bangladesh
The electronic data will be stored in a secured computer which can only be accessed with a secure password. Only the researchers will have access to the data.
All of your personal data including identifying information will be stored in a secure data-storing facility and secure servers for a minimum of 10 years following the end of the study at

The Initiative,
Main Office
Orange Grove Farm, Main Korung Rd
Banigala, Islamabad
Pakistan

AKR Foundation
Suite C-3 & C-4,
House #06,
Road # 109,
Gulshan-2, Dhaka-1212,
Bangladesh

Data will be transferred from Bangladesh and Pakistan to University of Edinburgh in the UK via a secure, password-protected online platform (DataSync) and then stored on a secure, password-protected DataVault / DataShare at University of Edinburgh for a minimum of 10 years following the end of the study. Your de-identified data will be shared with other researchers for future use. DataShare is an open access repository for anonymised data, which means that all non-identifiable data is freely available. DataVault is a secure repository for sensitive information which can only be accessed by approved researchers who have undergone a rigorous application and review process. After 10 years, all paper records will be shredded and disposed of securely and electronic records will be permanently erased. The data will be archived for up to 10 years on the University of Edinburgh server.
If you have any concerns about how we will use your information, please contact:

- The University of Edinburgh Data Protection Officer: dpo@ed.ac.uk
- Contact the Principal Investigator
  - Amina Khan, The Inititative, Main Office, Orange Grove Farm, Main Korung Road, Banigala, Islambad, Pakistan
  - Rumana Huque, ARK Foundation, Suite C-3 & C-4, House # 06, Road # 109, Gulshan-2, Dhaka-1212, Bangladesh

**8. How will the results be reported?**

The results of the trial will be published in international journals and presented at national and international meetings in both TB care and Tobacco Control. A lay summary of results will be distributed to local TB care networks. You will not be identifiable from any published results.

**9. What will happen if I want to stop?**

The project is voluntary so if you decide that you no longer want to carry on, you can stop at any time. You don’t have to give a reason if you do this and this will not affect your treatment at the hospital in any way or your legal rights. We would not ask for any further information but still like to use the information you have already provided in our research. However, if you specifically ask us not to use this information, we will delete this from our records. This will not affect your TB treatment. You will not be able to withdraw use of data from results that have already been published.

**10. What happens next?**

Any further questions you have about this study will be answered by the Principal Investigators:

Dr Amina Khan
The Initiative Islamabad Pakistan
Phone: 051-8732651
Email: aminakhan67@gmail.com.

Professor Rumana Huque
ARK Foundation, Bangladesh
Phone: +88 02 55069866
Email: rumanah14@yahoo.com

If you are ready to participate, we can continue now or if you wish to take 24 hours to consider participating in the study, we can schedule another appointment.

**11. Who do I contact if I have a problem or I have questions about the study?**
If you wish to discuss any aspect of the trial or have any concerns, the Principal Investigators are available to provide a complete explanation:

Dr Amina Khan
The Initiative Islamabad Pakistan
Phone: 051-8732651
Email: aminakhan67@gmail.com.

Professor Rumana Huque
ARK Foundation, Bangladesh
Phone: +88 02 55069866
Email: rumanah14@yahoo.com

**12. Independent Contact Details**
If you would like to discuss this study with someone independent of the study please contact

Dr Faiza Aslam
Director RGMO
Rawalpindi Medical University
Rawalpindi, Pakistan
Email: drfaizaaslam@gmail.com

Dr. Khaleda Islam
Technical Adviser
The Flemming Fund Bangladesh
Email: dr.khaleda.islam@gmail.com

**Thank you for taking the time to read this information sheet and considering if you will take part in this study.**

**Participant Consent Form (Adult)**
**<u>(Phase 4 mTB-Tobacco with Behavioral support)</u>**
**A mHealth intervention (mTB-Tobacco) for smoking cessation in people with tuberculosis: a two-stage adaptive design, randomised trial**
**Quit 4 TB Trial**

To be completed by the patient

If you agree with the statements please put an initial in the boxes below.

| | |
|---|---|
| 12. I confirm that I have read and understood the Participant Information Sheet for version v3.0 dated 28th March 2023 and have had the opportunity to consider the information, ask questions and have had these questions answered satisfactorily. | |
| 13. I understand that my participation is voluntary and that I am free to withdraw at any time, without giving any reason and without my medical care and/or legal rights being affected. | |
| 14. I understand that by taking part in this study my identity and location will be kept confidential. | |
| 15. I understand that the information I share and the data from my routine TB care will be accessed by the researchers. I give permission for these individuals to have access to my records. | |
| 16. I understand that data generated during the study will be sent outside of my home country to the United Kingdom, where laws protecting my personal information may be different to my own country. | |
| 17. I understand that de-identified data from this study may be shared with other researchers for future use. | |
| 18. I understand that my name will not be linked with the research materials, and I will not be identified or identifiable in any reports that result from the research. | |
| 19. I understand that if I withdraw from the study, the information I have already provided will still be used for the research. However, if I specifically ask you not to use this information, you will delete this from your records. | |
| 20. I agree to provide my complete contact details including CNIC number. | |
| 21. I understand that if I have concerns about this research, I can contact the Principal Investigator. | |
| 22. I agree to take part in the above titled study. | |

Participant's Full Name ____________________________________________________________
Date ___________________________________________________

| |
|---|
| Participant ‘s signature/thumb impression to be used if participant is illiterate: |
| Participant’s CNIC Number: ___________________________________________ |
| Participant’s Address<br>________________________________________________________________________________<br>________________________________________________________________________________<br>Telephone #: ____________________ |
| **Name of Person Obtaining Consent:**<br>Researcher Signature ___________________ Name of the Researcher_______________________<br>Date ___________________ |

**Name of Witness:**
Signed by impartial literate third party witness (*In case of illiterate Participant/ Legally Authorized Representative is illiterate*)

Signature:

Date: Place:

An NIHR Global Health Research Unit on Respiratory Health (RESPIRE) at the University of Edinburgh project.
***www.ed.ac.uk/usher/respire***
*This research was funded by the UK National Institute for Health and Care Research (NIHR) (Global Health Research Unit on Respiratory Health (RESPIRE); NIHR132826) using UK aid from the UK Government to support global health research.*
*The views expressed in this publication are those of the author(s) and not necessarily those of the NIHR or the UK Government*
