# Supplementary material for: mHealth intervention (mTB-Tobacco) for smoking cessation in people with drug-sensitive pulmonary tuberculosis in Bangladesh and Pakistan: protocol for an adaptive design, cluster randomised controlled trial (Quit4TB)
Source: BMJ Open. 2025 Feb 25;15(2):e089007. doi: 10.1136/bmjopen-2024-089007 (PMC12083406; doi:10.1136/bmjopen-2024-089007)
Supplement: Supplementary file 4 [file bmjopen-2024-089007.reviewer_comments.pdf]

PEER REVIEW HISTORY

BMJ Open publishes all reviews undertaken for accepted manuscripts. Reviewers are asked to complete a checklist review form and are provided with free text boxes to elaborate on their assessment. These free text comments are reproduced below.

ARTICLE DETAILS

Title (Provisional)

A mHealth intervention (mTB-Tobacco) for smoking cessation in people with drug-sensitive pulmonary tuberculosis in Bangladesh and Pakistan: Protocol for an adaptive design, cluster randomised controlled trial (Quit4TB)

Authors

Zahid, Maham; Rahman, Fahmidur; Chowdhury, Asiful; Rana, Shakhawat Hossain; Ansari, Saeed; Lim, Ai Keow; Danaee, Mahmoud; Boeckmann, Melanie; Parrott, Steve; Norrie, John; Khan, Amina; Huque, Rumana; Siddiqi, Kamran; Group, The RESPIRE

VERSION 1 - REVIEW

Reviewer 1

Name Bullen , Chris

Affiliation University of Auckland, National Institute for Health Innovation

Date 30-Nov-2024

COI I am on the International Scientific Advisory Committee for the RESPIRE research programme that funded the trial. I know several of the authors from conferences I have attended but have not published with them nor do I have any other formal connection with them.

Thank you for the opportunity to review this fascinating paper. The paper outlines a protocol for a moderately complex adaptive CRT design for a mobile phone-delivered text messaging intervention for TB patients in Pakistan and Bangladesh.

The rationale is well described.

The study will commence with a small PPI study, a small pilot, then the first CRT addressing the question of the superiority of the intervention over usual care; if found to be superior the next phase is a trial assessing the non-inferiority of the mCessation intervention over face to face counselling for quitting smoking.

The PPI study has a very small number of participants and may not capture the range of perspectives needed to ensure the intervention is going to fit with the health system and

patients' and their families' lives. There are few details provided about recruitment of this group.

The pilot study is not well specified and has the hallmarks of a feasibility study rather than a true pilot of the CRT outlined in the protocol.

The description of study methods is provided in detail and is of high quality and completeness as measured against the SPIRIT checklist.

The research team is very experienced in conducting large complex trials in these two countries with excellent collaborations with health services and I have little doubt they will be able to conduct and complete the planned studies to a high standard.

I have made a number of minor comments in the uploaded pdf comments, where there could be amendments to wording and more clarity on several matters (eg why are the economic endpoints not included as secondary outcomes? WIll smoking reduction be included as a secondary outcome? Will the analysis be for all types of smoked tobacco product collectively or dissagregated by type of product?)

The account of sample size estimation and statistical analysis is very good from my perspective as an epidemiologist but it would be helpful if a biostatistician with trials expertise could provide a statistical review.

Overall I thought this was a high-quality paper that meets the criteria for a protocol paper outlined by the journal and is well supported by the accompanying study materials.

The reviewer provided a marked copy with additional comments. Please contact the publisher for full details.

VERSION 1 - AUTHOR RESPONSE

Reviewer: 1

Dr. Chris Bullen , University of Auckland

Comments to the Author:

Thank you for the opportunity to review this fascinating paper. The paper outlines a protocol for a moderately complex adaptive CRT design for a mobile phone-delivered text messaging intervention for TB patients in Pakistan and Bangladesh.

The rationale is well described.

The study will commence with a small PPI study, a small pilot, then the first CRT addressing the question of the superiority of the intervention over usual care; if found to be superior the next phase is a trial assessing the non-inferiority of the mCessation intervention over face to face counselling for quitting smoking.

The PPI study has a very small number of participants and may not capture the range of perspectives needed to ensure the intervention is going to fit with the health system and patients' and their families' lives. There are few details provided about recruitment of this group.

Reply: Initially there were just two phases included in the protocol (superiority and non-inferiority), but later on as per suggestions of EMREC (ethics board of University of Edinburgh) the PPI and pilot phases were added, keeping in view the budget constraints, EMREC suggested to include few PPI members to have some insights on the study materials, and also asked to include a pilot phase to test feasibility of intervention and database.

The pilot study is not well specified and has the hallmarks of a feasibility study rather than a true pilot of the CRT outlined in the protocol.

Reply: You are right, its more like a small feasibility phase with ab object to test the feasibility/working of the mTB-Tobacco intervention portal to send auto-generated SMS messages to intervention clusters only, and RedCap database

The description of study methods is provided in detail and is of high quality and completeness as measured against the SPIRIT checklist.

The research team is very experienced in conducting large complex trials in these two countries with excellent collaborations with health services and I have little doubt they will be able to conduct and complete the planned studies to a high standard.

I have made a number of minor comments in the uploaded pdf comments, where there could be amendments to wording and more clarity on several matters (eg why are the economic endpoints not included as secondary outcomes? WIll smoking reduction be included as a secondary outcome? Will the analysis be for all types of smoked tobacco product collectively or dissagregated by type of product?)

Reply: Thank you for all the comments. I have tried to reply to each and every comment individually. Economic endpoints are included in the secondary outcomes, we missed to mention those, thanks for highlighting the same. Smoking reduction is not being captured as part of this trial. We plan to collectively analyse the quitting outcome for all types of smoked tobacco products including cigarettes, bidis and waterpipe sessions.

The account of sample size estimation and statistical analysis is very good from my perspective as an epidemiologist but it would be helpful if a biostatistician with trials expertise could provide a statistical review.

Overall I thought this was a high-quality paper that meets the criteria for a protocol paper outlined by the journal and is well supported by the accompanying study materials.
